# Bilateral Adnexal Entanglement: A Diagnostic Challenge in Postmenopausal Women

**DOI:** 10.7759/cureus.76802

**Published:** 2025-01-02

**Authors:** Maria Rui Torres, Luisa Cunha Silva, Rosália Coutada, Joao Pedro Prata, Paula Pinheiro

**Affiliations:** 1 Obstetrics and Gynecology, Unidade Local de Saúde do Alto Minho, Viana do Castelo, PRT; 2 Gynecologic Oncology, Unidade Local de Saúde do Alto Minho, Viana do Castelo, PRT; 3 Gynecology, Unidade Local de Saúde do Alto Minho, Viana do Castelo, PRT

**Keywords:** bilateral adnexal entanglement, diagnostic challenges, gynecological emergencies, mature cystic teratomas, ovarian masses, postmenopausal women

## Abstract

Simultaneous bilateral adnexal entanglement is a rare condition with only a few cases documented in the literature. It represents an exceptional cause of symptomatic pelvic masses, posing unique diagnostic and management challenges. A 56-year-old postmenopausal woman presented to the emergency department with progressive abdominal pain. Imaging revealed bilateral adnexal masses, initially interpreted as secondary lesions to prior abdominal surgeries. Surgical exploration uncovered bilateral adnexal entanglement without evidence of ischemia. Histopathology examination confirmed mature cystic teratomas. This case highlights the complexity of diagnosing adnexal masses in postmenopausal women, especially when imaging findings overlap with benign post-surgical changes. A thorough multidisciplinary approach is essential to avoid delays and ensure accurate diagnosis.

## Introduction

Adnexal torsion is a rare but significant gynecological condition, accounting for 2.7% of women presenting with adnexal masses. It is characterized by the twisting of ovarian or adnexal structures, potentially compromising vascular supply [1]. Risk factors include ovarian masses, hypermobility of adnexa, and prior pelvic surgery [2].

While unilateral adnexal torsion is the most commonly reported presentation, bilateral torsion is exceedingly rare, with fewer than 40 cases described in the literature [3]. Even more unusual is bilateral adnexal entanglement, where both adnexa become intertwined. This phenomenon can result from chronic adhesion formation due to previous interventions or abnormal anatomical positioning [4].

Bilateral adnexal entanglement presents unique diagnostic and management challenges, particularly in postmenopausal women, where adnexal pathologies are less frequent [5]. This rarity often leads to misdiagnosis, as gastrointestinal and urological conditions dominate the differential diagnosis for abdominal pain [6]. Additionally, in postmenopausal women, malignancy must always be considered when adnexal masses are identified, given the increased risk of gynecological cancers in this population [7].

The purpose of this article is to contribute to the limited literature on this condition, emphasizing the importance of maintaining a broad differential diagnosis, including less common gynecological etiologies, especially when imaging findings are ambiguous, and the need for a multidisciplinary approach to promptly manage such cases.

## Case presentation

A 56-year-old postmenopausal, nulliparous woman presented to the emergency department complaining of persistent lower abdominal pain for the last five days. She described the pain as gradually worsening, located to the lower abdominal quadrants, and radiating to the back. There were no associated symptoms such as nausea, vomiting, fever, or bowel habit changes. She also reported dysuria and urinary frequency, leading to an initial diagnosis of a urinary tract infection on a previous visit to the emergency department, four days before, for which she was treated with an outpatient course of antibiotics.

The patient’s medical history was significant for class 3 obesity, for which she underwent laparoscopic bariatric surgery in 2011, followed by abdominoplasty and thigh liposuction. Her daily medications included pantoprazole, sucralfate, and cholecalciferol. Menopause occurred at 42 years old, and she did not use hormone replacement therapy.

Upon evaluation in the emergency department, her vital signs were stable. Physical examination revealed mild tenderness in the lower abdomen without rebound tenderness or guarding. Initial blood tests showed hemoglobin levels of 11.4 g/dL, normal white cell counts without neutrophilia, and a slightly elevated C-reactive protein of 20 mg/dL.

**Figure 1:**
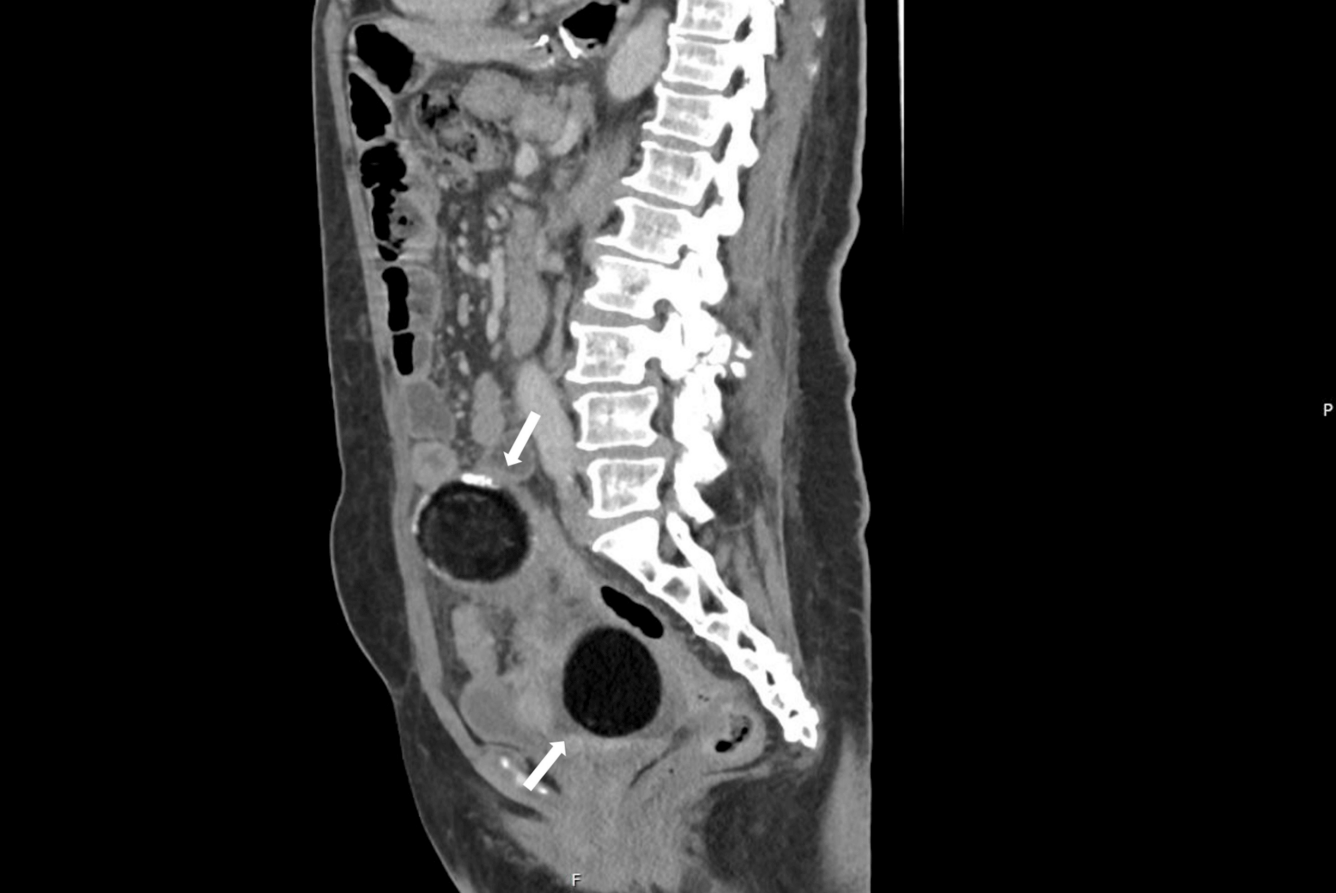
Abdominopelvic CT showing two adnexal masses (arrows), one of them in a much superior location in relation to the pelvis.

Given the persistence of symptoms despite antibiotic therapy, an abdominopelvic computed tomography (CT) scan was performed, revealing two complex masses apparently arising from the adnexa. The right adnexal mass measuring 86 × 76 mm, with marked fat density and surrounding soft tissue thickening, and the left adnexal mass, measuring 62 × 51 mm, appeared similar but lacked inflammatory findings, as shown in Figure 1. These images were compatible with bilateral teratomas, though the differential diagnosis included post-surgical steatonecrosis or lipomatous tumors secondary to prior surgical interventions, as these lesions had been previously described in a CT scan from 2021 with no further investigation lead on as they had no associated symptomatology [2].

Observation by Gynecology was requested and pelvic examination revealed a regular, mobile, non-tender cervix and no palpable masses in adnexal topography. Transvaginal ultrasonography identified a heterogeneous cystic mass measuring 8 cm posterior to the uterus, with a reticular pattern suggesting ovarian stroma, color score 1, but the second mass was not visualized with this imaging modality due to its higher abdominal position. Tumor markers were obtained, showing mildly elevated cancer antigen (CA) 125 (45.3 U/mL) and CA 19-9 (406.56 U/mL), as described in Table 1. The Calculation of the Risk of Malignancy Index revealed a low risk for malignancy, with a score of 135 points.

**Table 1:** Relevant laboratory findings.

Tests	Result	Normal range
Hemoglobin	11.4 g/dL	11.5–15.5 g/dL
White blood cell count	7,000/mm^3^	4,500–11,000/mm^3^
Neutrophils	58%	54–62%
C-reactive protein	20 mg/dL	<0.5 mg/dL
Cancer antigen 125	45.3 U/mL	0–35 U/mL
Cancer antigen 19-9	406.56 U/mL	0–37 U/mL

**Figure 2:**
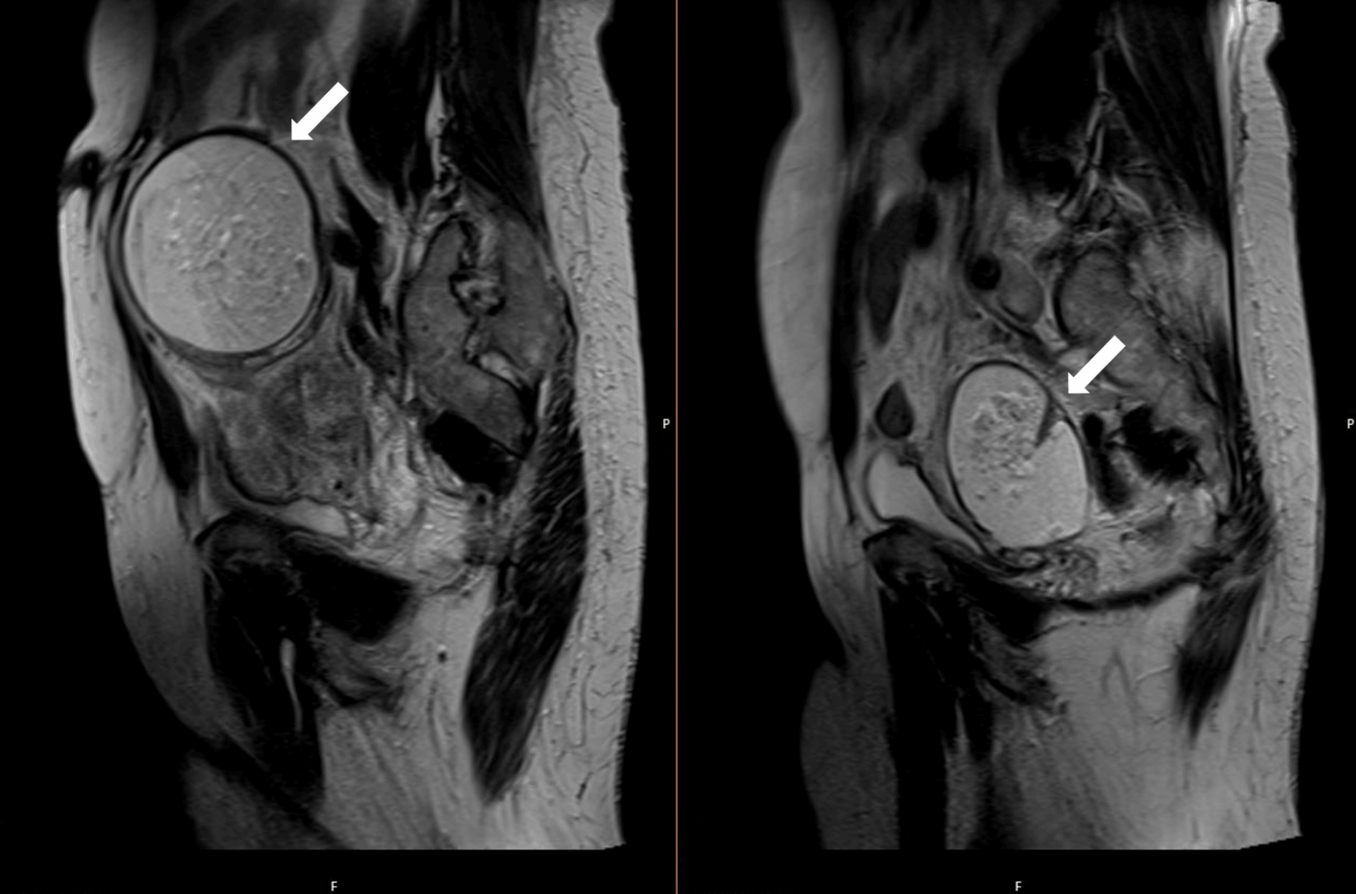
Sagital T2 MRI images of the pelvis showing bilateral adnexal masses (arrows) with predominant fatty components and internal nodular areas (hyperintense).

**Figure 3:**
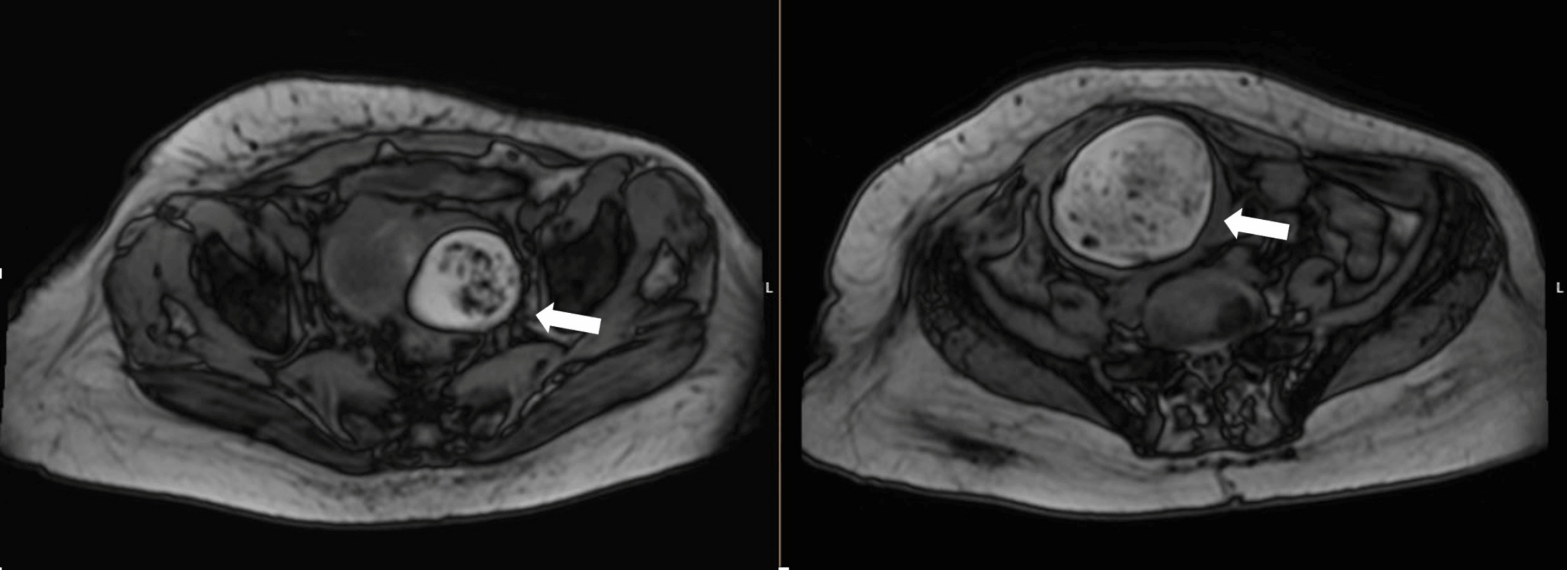
Axial MRI scan of the pelvis showing bilateral adnexal masses (white arrows) with predominant fatty components.

**Figure 4:**
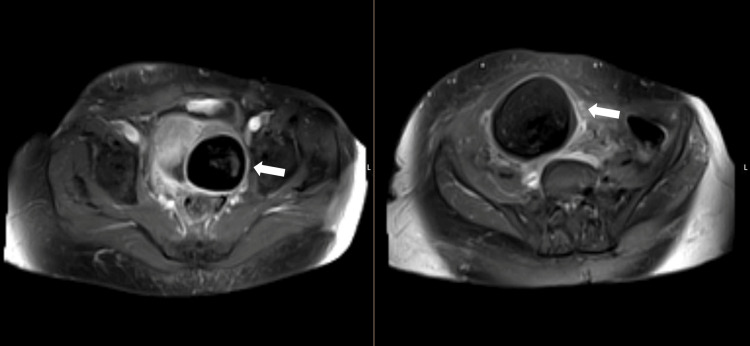
Axial MRI scan of the pelvis after gadolinium injection showing the enhancing component (arrows).

To better characterize the adnexal masses, pelvic magnetic resonance imaging (MRI) was performed, confirming the presence of bilateral adnexal masses with predominant fatty components and internal nodular areas enhancing with gadolinium, as shown in Figures 2-4. These findings supported the diagnosis of mature cystic teratomas without evidence of malignancy or ischemia.

**Figure 5:**
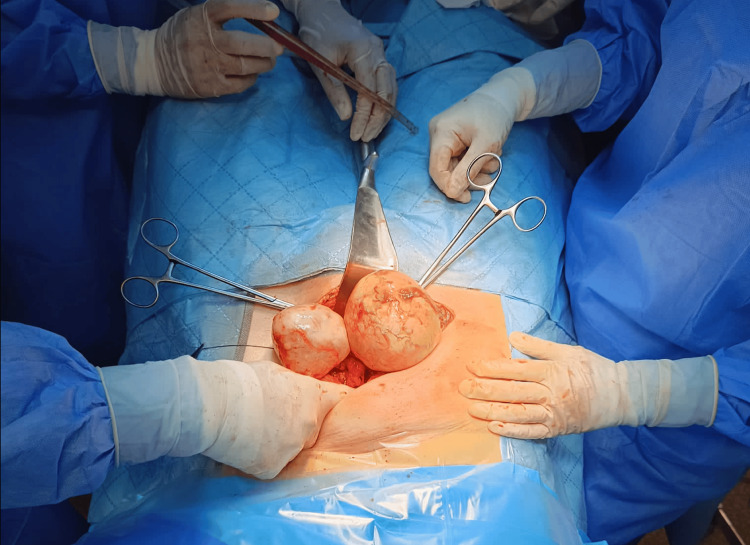
Upon surgical inspection, both adnexa were intertwined and placed up in the abdominal cavity, far from their typical anatomical position.

**Figure 6:**
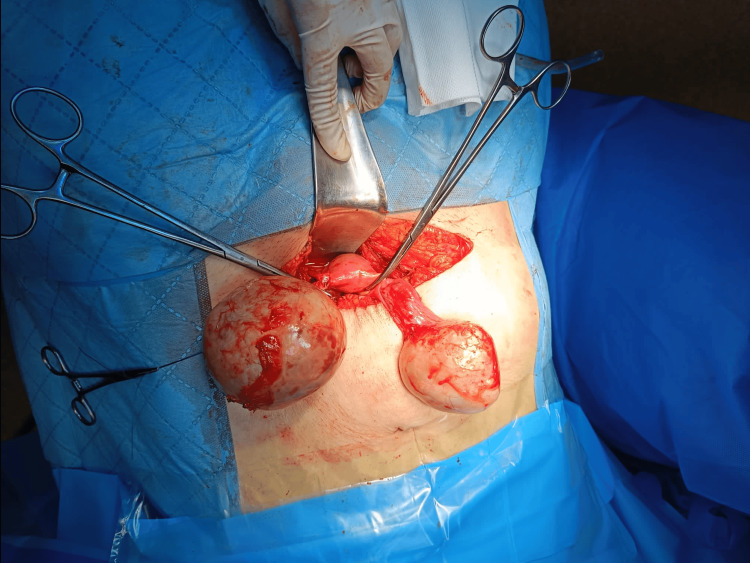
After untangling the adnexal masses, both adnexa were vascularized and no ischemic changes were observed.

Despite the absence of acute ischemic signs, the persistence of symptoms and elevated tumor markers prompted a multidisciplinary discussion, and the patient was scheduled for an exploratory laparotomy. During surgery, two adnexal masses were identified: one in the right adnexa (measuring 8 cm) and another in the left adnexa (measuring 6 cm). Both were interconnected by thin adhesions, forming an entanglement that placed them far from their typical anatomical position. Despite the abnormal anatomical configuration, both adnexa were vascularized and no ischemic changes were observed (Figures 5, 6). The uterus appeared normal for the patient’s age. A bilateral salpingo-oophorectomy was performed, and the masses were sent for frozen section analysis, which confirmed mature cystic teratomas.

Histopathological examination corroborated the intraoperative findings, describing mature cystic teratomas in both adnexa without malignant transformation. After surgery, the patient recovered uneventfully, experiencing complete resolution of abdominal pain. She was discharged from follow-up after a postoperative evaluation confirmed her stable condition.

## Discussion

This case highlights a very rare entity: bilateral adnexal entanglement. Only a few cases are described in the literature and bilateral entanglement without torsion, as seen in this clinical case, is even rarer [8-10]. The involvement of both adnexa adds complexity, particularly in postmenopausal women, where this diagnosis is less frequently suspected, as ovaries tend to be smaller [3] and ovarian masses are usually linked to malignancy suspicion.

The differential diagnosis of adnexal masses and abdominal pain in postmenopausal women can be complex as there are several possibilities, particularly when imaging findings are ambiguous [5]. The initial CT findings described the masses as consistent with lipomatous lesions or areas of steatonecrosis, likely secondary to the patient’s history of bariatric surgery and subsequent abdominoplasty. Steatonecrosis is a known complication of prior abdominal surgery and is often misinterpreted as post-inflammatory changes on imaging [6]. This reassuring finding can lead to delays in identifying adnexal pathologies, as evidenced in this case, where initial vague symptoms of progressive abdominal pain and urinary symptoms were linked to a much more usual pathology such as urinary tract infection. Subsequent MRI was critical in identifying bilateral masses with characteristics consistent with mature cystic teratomas. However, these findings did not raise the suspicion of adnexal entanglement, illustrating the limitations of imaging in such rare cases.

The mild clinical presentation, with an absence of acute ischemic signs such as severe pain or systemic inflammatory response, further distinguishes adnexal entanglement from typical adnexal torsion, emphasizing the need for heightened clinical suspicion in atypical scenarios [2,6]. These, as well as the slightly elevated tumor markers and the benign-appearing imaging findings, precluded the need for emergent surgical intervention. However, the development of persistent clinical symptoms and the need to exclude malignancy at such age [5] warranted surgical exploration that not only allowed the gold-standard method for the definitive diagnosis but, in this case, was also crucial for symptom relief. The intraoperative finding of bilateral adnexal entanglement surprised the surgeons as this scenario had not yet been considered due to its rarity. Despite the abnormal anatomical position due to the twisting of adnexal structures placing them higher in the abdominal cavity, both were still vascularized, and no signs of torsion or malignant transformation were found.

Histopathological confirmation of mature cystic teratomas, also known as ovarian dermoid cysts, highlighted the benign nature of the lesions. They are the most common type of germ cell tumors in the ovary, with a reported incidence of 1.2 to 14.2 cases/100,000 per year, especially in reproductive age. The presence of teratomas in both adnexa is itself rare, occurring in 10-15% of cases. As described in this case, these tumors are typically benign, with an estimated risk of malignant transformation of 0.7 to 2% [1,4], most often into squamous cell carcinoma.

This case underscores the challenges of diagnosing adnexal entanglement on imaging, as this rare anatomical configuration is often not evident preoperatively, even when the characteristics of the masses themselves are suggestive of benign lesions such as teratomas. It also highlights the importance of a multidisciplinary approach including gynecology, radiology, and oncology in the early recognition of this entity to guide timely surgical intervention, preventing complications such as irreversible ischemic damage.

## Conclusions

The presented case highlights the diagnostic challenges associated with identifying adnexal entanglement in postmenopausal women, as this rare anatomical finding is often not apparent on imaging. Clinicians should be aware of this condition in the differential diagnosis of adnexal masses and abdominal pain, even when the masses themselves have reassuring benign features, especially in patients with prior abdominal surgery, after ruling out the most common causes. Malignancy must always be considered in postmenopausal women, implying a detailed investigation. Proper clinical evaluation and surgical intervention are crucial for accurate diagnosis and prevention of irreversible damage.
